# An Experimentally Benchmarked Optical Study on Absorption Enhancement in Nanostructured a-Si/PbS Quantum Dot Tandem Solar Cells

**DOI:** 10.3390/nano16010012

**Published:** 2025-12-21

**Authors:** Qinqian Jiang, Zeyu Li

**Affiliations:** 1 School of Integrated Circuits, Nanjing University of Information Science and Technology, Nanjing 210044, China; 202383270425@nuist.edu.cn; 2 School of Computing, Engineering & Mathematical Sciences, La Trobe University, Melbourne, VIC 3086, Australia

**Keywords:** tandem solar cell, nanopillar grating, broadband absorption enhancement

## Abstract

Tandem solar cells offer a promising route to surpass single-junction efficiency limits. The amorphous silicon (a-Si)/lead sulfide quantum dot (PbS QD) configuration is a strong candidate for broadband solar spectrum utilization. Planar devices with this material combination suffer from significant optical losses, making advanced light management essential. To address this, we propose a novel experimentally guided nanostructure design. Our proposed method utilizes nanostructures to increase the optical path length by diffracting light to off-normal directions and employs graded-index material stacks to suppress surface reflectance. This work establishes a clear design pathway and provides valuable insights into alternative light management strategies for the future commercialization of these tandem solar cells.

## 1. Introduction

The efficiency of conventional single-junction solar cells is fundamentally constrained by the Shockley–Queisser limit, as a single absorber cannot effectively harvest photons across the full solar spectrum [1]. Tandem solar cells surpass the single-junction limit via spectral splitting: a wide-bandgap top subcell absorbs high-energy photons and transmits lower-energy photons to a narrow-bandgap bottom subcell for harvesting [2].

Recently, lead sulfide quantum dots (PbS QDs) have attracted considerable attention as a promising absorber for the bottom subcell in tandem architectures, owing to their size-tunable bandgap and strong response to long-wavelength photons [3,4,5]. However, PbS QDs exhibit notable limitations at shorter wavelengths, including weak absorption and pronounced photochemical instability, particularly under ultraviolet (UV) illumination [6]. Moreover, although reducing the QD size can enhance absorption in the visible region, it often results in a lower open-circuit voltage (Voc) due to increased surface defect density and recombination losses [7]. To address these limitations, amorphous silicon (a-Si) has emerged as a promising top-cell material [8]. As a mature photovoltaic absorber, a-Si exhibits strong absorption in the blue and green regions of the solar spectrum [9], complementing the long-wavelength absorption of PbS QDs in the bottom cell [3].

Despite its promise, the performance of planar tandem architectures is notably constrained by several intrinsic optical limitations. A pronounced refractive index mismatch between the high-index a-Si top subcell and the lower-index PbS QD bottom subcell induces significant interfacial reflection losses, thereby impeding current generation in the bottom subcell [9,10,11]. Furthermore, the device is limited by parasitic absorption within intermediate electrode layers (e.g., ITO) [10,12,13,14], high surface reflectance from the top subcell [10,15], and detrimental destructive interference effects within the multi-layer stack [9,11]. Collectively, these optical losses constitute a major impediment, hindering the full realization of the material system’s potential. Therefore, advanced photon management strategies are essential for performance enhancement, with nanostructures emerging as a promising approach for effective light trapping [16,17,18].

Nanostructured light management techniques are widely employed for light trapping. Nanopillars [19], nanowires [20], and nanocone arrays [21] are common architectures for light management [22]. While these structures can significantly enhance absorption, they also exhibit notable challenges, including wavelength-specific absorption enhancement rather than broadband absorption [23], inherent parasitic losses due to material mismatch [24], and lack of experiment robustness due to stringent requirements for coherence and phase control [25]. To address these limitations, our work focuses on a specific grating-based architecture with special considerations. We term this design the Nano Concentric Circular Nanopillar Grating (NCCNG). Our design facilitates advanced photon management by simultaneously suppressing surface reflection, mitigating parasitic absorption, and extending the optical path length to enhance overall absorption [20]. First, high surface reflectance is mitigated by a graded effective refractive index formed at the top interface, which serves as a highly efficient anti-reflection layer to suppress reflection losses [15]. Second, the dual challenges of wavelength-specific enhancement and stringent coherence requirements are simultaneously resolved. The architecture functions as a powerful diffraction grating, scattering incident light into large-angle propagation modes [26]. This mechanism disrupts detrimental interference effects and extends the optical path length, thereby promoting the desired broadband absorption [25]. Finally, the design addresses the critical challenge of experimental robustness by providing superior mechanical stability compared to the tall, fragile nanowire arrays [27,28].

This study employs a systematic simulation approach [29], where the optical simulation model was first rigorously guided and calibrated against experimental data [30] to accurately replicate experimental outcomes. Based on the simulation results, the dominant sources of optical loss in the planar tandem solar cell were systematically identified [31], leading to the proposal of the NCCNG structure to verify its optical performance via simulation. Crucially, this investigation focuses exclusively on the optical absorption limits and light-trapping mechanisms within the tandem material stack. While the physical layer sequence mimics a monolithic tandem configuration, detailed electrical characterizations—such as photocurrent matching, series-connection efficiency, and open-circuit voltage estimations—are not modeled in this work, as the rigorous methodology for such electrical metric estimations was established in our previous study [32]. Consequently, the analysis presented here is strictly limited to optical absorptance enhancement. This work not only addresses limitations in light management of traditional structures, but also provides a robust, experimentally guided framework for future tandem solar cells with different material combinations [33,34].

## 2. Materials and Methods

The electromagnetic simulations were performed using Ansys HFSS based on the Finite Element Method (FEM). Detailed numerical protocols—specifically the mesh strategy, Perfectly Matched Layer (PML) boundary conditions, and convergence criteria—follow the rigorous simulation framework established in our previous work [32]. Regarding the environmental context, the model assumes a standard air background (n=1) above the top contact and a semi-infinite substrate below to strictly define the radiation boundaries.

To establish a realistic optical model, the simulation parameters for the PbS QD absorber were strictly benchmarked against experimental spectroscopic ellipsometry data from Zhang et al. [30]. Specifically, the model replicates the optical properties of a PbS QD (approx. 3.5 nm diameter) solid film passivated with TBAI ligands, ensuring an accurate representation of the material’s dispersion and absorption characteristics. These extracted optical constants were further cross-referenced with recent studies on high-efficiency PbS devices [35,36,37,38], confirming their consistency with state-of-the-art experimental systems.

**Figure 1:**
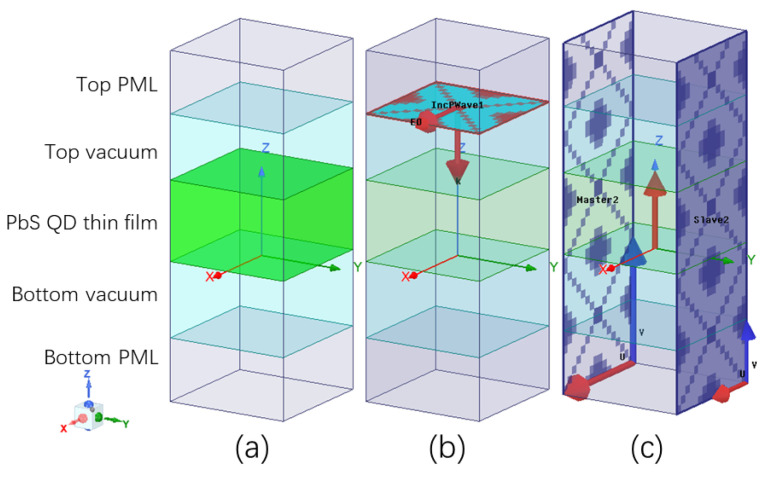
(**a**) The layered structure of the simulation unit cell; (**b**) the plane wave excitation source setup; and (**c**) the periodic boundary condition setup.

The accurate reconstruction of $\epsilon^{\prime }$ and tanδ from n and k allows correct EM modeling of the corresponding material system in HFSS [21,39]. Figure 1 shows details of the simulated unit cell of the planar PbS QD thin film. The layers in the model are the top perfect matching layer (PML), top vacuum, PbS QD thin film, bottom vacuum, and bottom PML. As shown in Figure 1b, an EM plane wave excitation source was placed above top vacuum, with the E field aligned to hte +x axis and wave propagation along the −z axis. Due to the geometrical symmetry of our models in this paper, TM/TE modes are not discussed separately [40,41]. As shown in Figure 1c, two pairs of master–slave planes were placed in opposite directions at the four sides of the unit cell. This allows for a continuous boundary condition of the opposite faces. As the result, an infinitely large PbS QD thin film in vacuum could be simulated. The simulated final results would be comparable with experimental data, considering that light spot size is much smaller than sample size in any common spectrometer setup.

**Figure 2:**
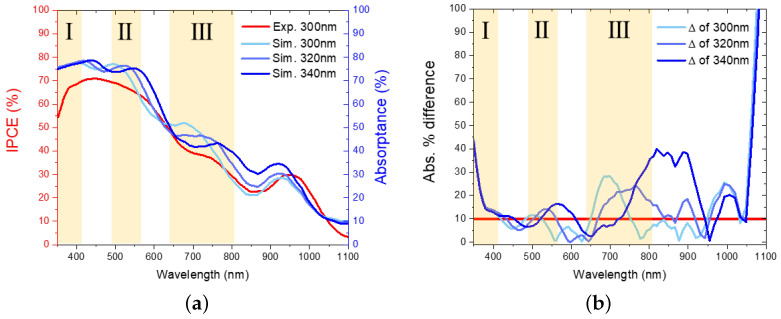
(**a**) Comparison of the simulated absorptance spectrum with the experimental IPCE spectrum; (**b**) the percentage difference between the simulated and experimental results.

After that, absorptance spectra of the planar PbS QD thin films with different thicknesses were simulated. And comparisons with the measured result from the same reference [30] were carried out. Figure 2a shows the experimental incident photon-to-current efficiency (IPCE) of the 300 nm PbS QD thin film from ref. [30] (reprint with permission) and our simulated absorptance of 300, 320, and 340 nm thick PbS QD thin films. The simulated absorptance spectra agree reasonably well with experimental IPCE spectra, with the exception of certain regions. To better illustrate this, Figure 2b shows the absolute value of percentage difference between simulated absorptance and experimental IPCE. The red line indicates a threshold of 10%, above which we suggest simulation results significantly deviate from experiment results. Such deviations stem from several effects. Firstly, for wavelengths below 560 nm, i.e., in region I and II, the experiment shows an underestimation compared to the simulation. Such additional parasitic losses observed in the experiment might be introduced by nanoscale surface roughness and defects, where the former induces undesired light scattering for blue and green light [25] and the latter leads to reduced current collection efficiency [25,30,42]. Secondly, the PbS QD thin film might not be accurately parallel and homogeneous during fabrication. As in region II and III, simulated absorptance peaks red shift with increasing film thickness, which suggests an interference effect. However such peaks are not observed in the experiment result in region II due to pre-stated surface influence. The shouldered peak shifting in region III could be confirmed by the similar side peak of the experiment result when comparing with the simulated 320 nm shown in Figure 2a. Based on this interpretation, we observed that unlike a continuous worse-off deviation with increasing film thickness in region II, in region III, the simulated 320 nm deviates more broadly in wavelength range but less in percentage value when compared to the simulated 300 nm. Such inconsistency might be due to the above-stated second reason as well. Thirdly and lastly, n and k measurement by ellipsometry might be inaccurate beyond 850 nm, while approaching the bandgap of the PbS QD thin film [43,44,45]. As in the 900–1000 nm wavelength range, an absorptance peak offset of around 30nm was observed between simulation and experiment results.

**Figure 3:**
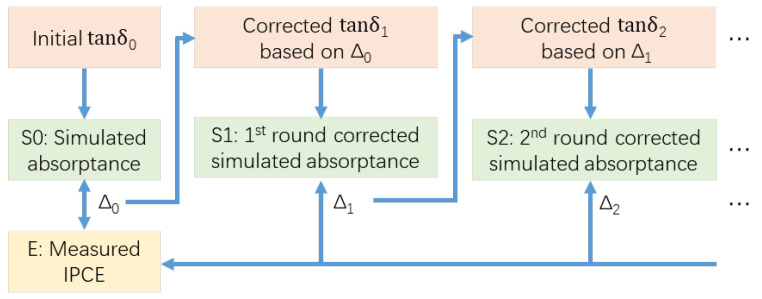
Flowchart of the point-by-point (PBP) iterative fitting procedure.

Taking into consideration the above plausible effects, to improve the accuracy of the model, we carried out a point-by-point (PBP) iterated fitting of the PbS QD thin film dielectric loss tangent at a fixed thickness of 300 nm. Since the dielectric loss tangent is a measure of the energy dissipation rate while an EM wave travels through the material, by fitting it to an effective value, one might be able to include additional factors that affect the actual absorptance. Therefore, simulated results could finally be accurately benchmarked against experimental results [46]. Figure 3 shows the flowchart of the procedure. The difference between simulated and experimental results was used to correct the dielectric loss tangent for the next round of simulation, using the formula below:

(1) $$\begin{array}{cc} \Delta_{n}\left(\lambda \right) & =\frac{s_{n}\left(\lambda \right)}{E(\lambda )} \end{array}$$

(2) $$\begin{array}{cc} \tan\delta_{n+1}\left(\lambda \right) & =\frac{\tan\delta_{n}\left(\lambda \right)}{SF^{\left(\Delta_{n}\left(\lambda \right)-1\right)}} \end{array}$$

where *n* represents the current iteration number, $S_{n}\left(\lambda \right)$ denotes the simulated absorptance spectrum at the *n*-th iteration, and E(λ) represents the experimental IPCE benchmark spectrum. The term $\Delta_{n}\left(\lambda \right)$ is the calculated deviation ratio used to update the material properties. The scaling factor (SF) was chosen to be 10 to balance between the calculation accuracy and computational efficiency.

**Figure 4:**
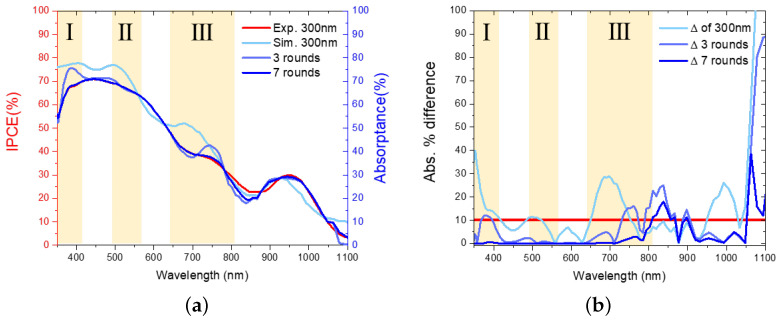
(**a**) The convergence of the simulated spectrum to the experimental spectrum during the fitting process; (**b**) The percentage difference between simulated and experimental results. The yellow shaded areas I–III represent spectral regions dominated by parasitic losses (I and II) and thickness-dependent interference effects (III).

Figure 4a shows the experiment, initial simulation, and corrected simulation results after three and seven rounds of fitting. Figure 4b explicitly quantifies the fitting error, serving as the convergence metric for the PBP iteration process. The final model achieves an average spectral deviation of less than 5% across the relevant solar spectrum, demonstrating the rigorous convergence of our calibration method and validating the accuracy of the extracted parameters. A detailed sensitivity analysis justifying the validity of this point-by-point inversion approach is provided in Supplementary Note S1.

**Figure 5:**
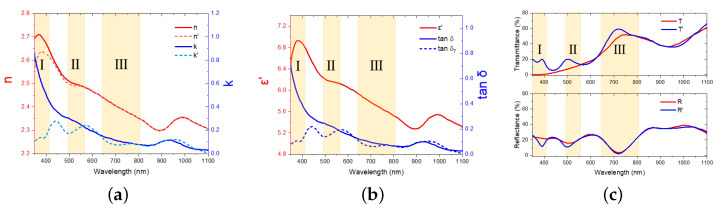
(**a**) The final calibrated effective refractive index (n’) and extinction coefficient (k’); (**b**) the corresponding effective real part of the complex relative permittivity ($\varepsilon^{\prime }$) and dielectric loss tangent ($\tan\delta^{\prime }$) extracted from the experimental baseline of Zhang et al. [30] (reprint with permission); and (**c**) the reflectance (R’) and transmittance (T’) calculated using the effective optical constants. The yellow shaded areas I–III represent spectral regions dominated by parasitic losses (I and II) and thickness-dependent interference effects (III).

Figure 5a shows the fitted effective refractive index n’ and extinction coefficient k’ solved from the real part of relative permittivity $\epsilon^{\prime }$ and the corrected dielectric loss tangent $\tan\delta_{7}$, shown in Figure 5b. Figure 5c shows the corrected reflectance R’ and transmittance T’. Comparing to their initial values, both the refractive index and extinction coefficient were significantly reduced in region I and II and were slightly corrected in region III and beyond. As the result, reduced reflectance and enhanced transmittance were observed in region I and II, shown in Figure 5c. Referring to previously mentioned nanoscale roughness on the sample surface, such conditions will indeed reduce the reflection due to light trapping and enhance transmission due to forward light scattering [46], consistent with findings reported in the literature [47].

Summarizing the discussion above, we suggest using the effective n’ and k’ as the PbS QD thin film material parameter for further investigation. Hence, we refer to seven rounds of fitting as the benchmarked baseline simulation. Calibrating the “effective optical constant” model against IPCE [30] relies on specific physical assumptions. We postulate that the macroscopic effects of surface roughness—comprising both parasitic light scattering [25] and surface defect-induced carrier recombination [30,42]—can be approximated by tuning the dielectric loss tangent of the planar film [46]. Because IPCE convolves optical absorptance with carrier collection efficiency [30], this fitting process embeds electrical losses directly into the extracted parameters. A recognized limitation of this approach is the inability to decouple optical scattering [25] from electrical recombination [42]; rather, the cumulative reduction in quantum efficiency is modeled as effective optical attenuation [46]. Nevertheless, this approximation yields a conservative simulation baseline. Optimizing the nanostructure using these penalty-inclusive parameters ensures the robustness of the reported absorption enhancement. In a purely optical regime where electrical losses are excluded, the intrinsic absorption efficiency would theoretically surpass these conservative simulations. Future work will focus on validating these constants across varying film thicknesses to further resolve the optical and electrical contributions.

## 3. Results and Discussion

### 3.1. Planar Tandem Device

**Figure 6:**
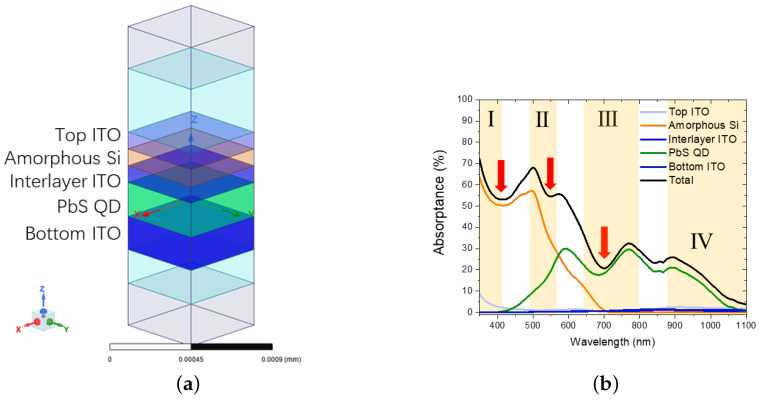
(**a**) Schematic of the planar tandem device structure; (**b**) the absorptance spectra of each layer and the total device, with optical bottlenecks indicated. The red arrows indicate the optical bottlenecks caused by destructive interference within the multilayer stack. The yellow shaded areas I–IV highlight specific spectral regions used to analyze parasitic losses (I and II) and interference-driven absorption fluctuations (III and IV).

In the previous part, we benchmarked the optical parameters of the PbS QD thin film. In this part, we will investigate the optical performance of the tandem solar cell device. Figure 6a shows the planar device structure unit cell. The planar device architecture, arranged in descending order from the top surface, comprises a 100 nm top ITO contact, a 100 nm amorphous silicon (a-Si) thin film, a 100 nm interlayer ITO for carrier transport, a 200 nm PbS QD thin film, and a 200 nm bottom ITO contact. The optical constants for the ITO layers were adopted from König et al. [48]. Thinner amorphous Si and PbS QD films were chosen to achieve current matching [9,31,49,50] and also to avoid increasing carrier recombination when films were made thick [50,51]. Due to the wider bandgap of amorphous Si compared to PbS QDs, we designed amorphous Si on top, as the top subcell for shorter-wavelength absorption, and PbS QDs at the bottom, as the bottom subcell for longer-wavelength absorption.

With the help of HFSS, we are able to investigate the absorptance of each layer separately. Figure 6b shows the simulated absorptance results. We observed that the 100 nm top cell can effectively absorb a shorter wavelength range up to 550 nm [52]. Beyond this, absorptance of the 200 nm bottom cell increases effectively and covers the rest of the solar spectrum all the way to 1100 nm. Parasitic absorptance of ITO was found to be at a low level for most of the spectrum, typically around 5%, for example, in region IV. However, in region I, top ITO contact parasitic losses were significant [12,13,14]. In our simulation of the planar tandem device based on the calibrated optical model, we have identified this significant parasitic loss in the top ITO layer as a primary optical bottleneck. We would expect even worse parasitic losses in actual experiment results considering the nano roughness surface condition.

Even though we constructed a tandem device with an appropriate film arrangement and comparable active layer thickness, the optical performance is not improved compared to the 300 nm PbS QD thin film alone, referring to Figure 4a. Other than parasitic absorptance of ITO, several other factors were also suspected. Firstly, due to a higher permittivity, reflectance of the amorphous Si is higher than that of the PbS QD film. Light shining from the top of the device will be reflected more significantly, and less energy will be collected by the bottom subcell [15]. Such an effect can be observed from the overall reduced total absorptance spectrum. Secondly, due to interferences in the designed multiple thin film stack system, destructive interferences were introduced at 350–490 nm, 520–560 nm, and 610–760 nm ranges, as shown with red indicators [9]. Absorptance was reduced, especially near the first and last indicators [25]. Thirdly, optimization of the film thicknesses of either amorphous Si or the PbS QD film was difficult due to the lack of a reference. Hence, due to these complex and competing optical effects, achieving an optimal solution merely by tuning the film thicknesses is exceedingly difficult [53]. These significant optical losses inherent in the planar configuration highlight the need for a more advanced device architecture based on nanophotonic principles.

### 3.2. Nanostructured Tandem Device

To address the optical limitations of the planar device, we designed and simulated a nanostructured tandem solar cell.

**Figure 7:**
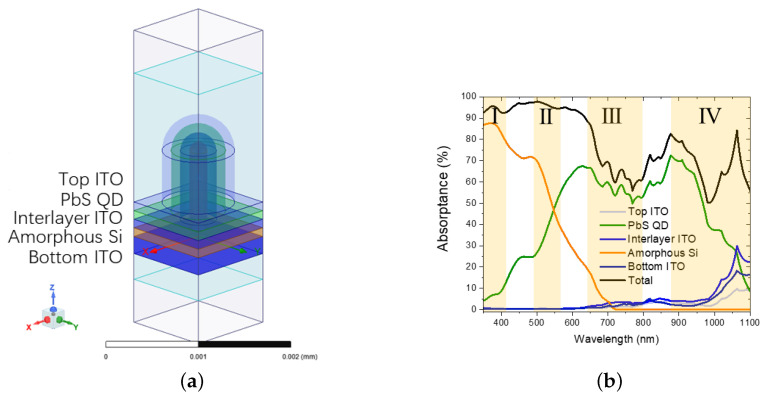
(**a**) Schematic of the nanostructured tandem device structure; (**b**) the absorptance spectra of each layer and the total device under normal incidence.The yellow shaded areas I–IV highlight specific spectral regions used to analyze parasitic losses (I and II) and interference-driven absorption fluctuations (III and IV).

Figure 7a illustrates the 3D unit cell of the Nano Concentric Circular Nanopillar Grating (NCCNG). The structure is periodic, defined by an array periodicity (*P*) of 500 nm and a central core radius (*R*) of 100 nm. It features a quasi-3D topology established via conformal patterning, where the hemispherical cap creates a graded effective refractive index profile to physically minimize surface reflection and enable broadband absorption [23]. The layer sequence, arranged from bottom to top, comprises a 150 nm planar bottom ITO, 100 nm a-Si:H, 100 nm interlayer ITO, 100 nm PbS QD, and 100 nm top ITO. This architecture is rigorously optimized for the fabrication of the a-Si/PbS system, justifying the distinct layer configurations employed. While the planar reference utilizes an “a-Si on PbS” sequence to maximize optical spectral splitting [8], the nanostructured device adopts an inverted “PbS on a-Si” architecture. This inversion is dictated by thermal budget compatibility: the central a-Si nanopillars must be grown first via the high-temperature bottom-up PECVD mechanism of radial junctions [51], whereas the concentric grooves accommodate the subsequent infiltration of temperature-sensitive PbS QD ink via spin-coating [54]. Consequently, this design benchmarks the “fabrication-viable” nanostructure against the “optically optimal” planar baseline, establishing a rigorous standard for performance evaluation [51]. To ensure a rigorous comparison between the planar and nanostructured architectures, we adhered to the principle of “equivalent material usage per unit area”. While the geometric thicknesses of the active layers in the nanostructured device inherently differ from those of the planar counterpart due to the quasi-3D topology, the total volume of the absorber materials (a-Si and PbS) is kept identical across both designs. This methodology aligns with practical experimental conditions where deposition duration is kept constant, a standard practice established in prior studies [51]. By strictly controlling for material volume, we ensure that the observed performance enhancements are attributed exclusively to the advanced light management capabilities of the nanostructure, rather than to an increase in the absorber material mass. The simulated absorption results in Figure 7b demonstrate a significant performance enhancement. The total absorption curve is smooth and free of sharp dips. It consistently exceeds 80% and peaks at over 95% throughout the visible spectrum. Furthermore, parasitic absorption in all ITO layers is suppressed to negligible levels [24,52]. This work, therefore, provides an effective design to overcome key optical bottlenecks. It also offers a clear pathway for future performance optimization [33,55].

**Figure 8:**
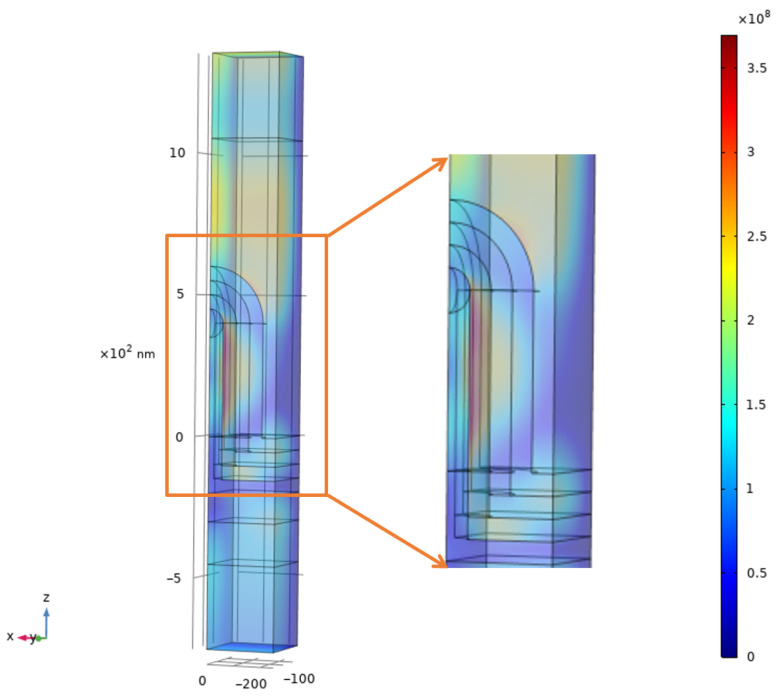
Volume electric field intensity (|E|) distribution of the nanostructured tandem device at λ=800 nm under normal incidence. A one-quarter cut of the 3D unit cell is presented, with symmetry boundary conditions applied in the simulation. Key features include (1) strong field localization (’hotspots’) at the nanopillar interfaces, confirming the efficient light coupling mechanism enabled by nanophotonic scattering resonances; (2) the re-emergence of high-intensity regions deep within the absorber layer, providing direct evidence of diffraction-induced optical path length extension; and (3) a smooth field transition from air into the device with minimal standing wave patterns above the structure, indicating the effective suppression of surface reflection and destructive interference.

The observed performance enhancement arises from the synergistic interplay of three optical mechanisms, explicitly substantiated by the electric field intensity (|E|) profiles in Figure 8 and the absorptance spectra in Figure 7b. First, parasitic absorption is mitigated; the pronounced field localization at the nanopillar interfaces confirms an efficient light coupling mechanism enabled by nanophotonic scattering resonances, which acts to concentrate incident light into the active layers [24,52]. This mechanism efficiently channels incident photons into the active absorbers, yielding the negligible parasitic losses within the ITO layers observed in Figure 7b. Second, surface reflection is suppressed through a graded effective refractive index. The field map reveals a seamless optical transition from the ambient medium into the device, characterized by the absence of standing wave patterns above the structure. This graded effective refractive index profile effectively minimizes back-reflection, directly underpinning the substantial enhancement in total absorptance shown in Figure 7b. Finally, the architecture induces optical path length extension and suppresses destructive interference. The field distributions demonstrate that incident light is diffracted into oblique propagation modes, enabling high-intensity fields to re-emerge deep within the absorber volume. This effective extension of the optical path length directly accounts for the significant broadband absorption enhancement observed in Figure 8. Furthermore, this scattering mechanism disrupts coherent interference fringes—an effect most pronounced in the PbS QD layer. This spatial homogenization of the optical field effectively fills the destructive “dead zones”, resulting in the smooth, broadband absorption profile presented in Figure 7b.

It should be noted that the results presented above were calculated under normal incidence conditions. Nevertheless, we anticipate that the NCCNG architecture will exhibit significant robustness against variations in the angle of incidence (AOI). Unlike planar films, which are sensitive to interference effects at oblique angles, the quasi-3D topology and the resulting graded refractive index profile naturally facilitate omnidirectional light coupling. This angular insensitivity is consistent with our previous investigations on similar nanostructured arrays [32], suggesting that the high absorption performance would be maintained over a broad range of incident angles.

## 4. Conclusions

In this work, we first established an optical model for PbS QDs through rigorous benchmarking and calibration against published experimental data. Based on this model, we then designed and systematically analyzed the optical characteristics of a planar a-Si and PbS QD tandem solar cell. Our analysis reveals that the optical absorption efficiency of the planar tandem device is severely constrained by three key factors: parasitic absorption loss from the ITO layers; high surface reflectance from the amorphous Si top cell; and destructive interference arising from the multi-layer stack. To overcome these optical bottlenecks, we propose a novel nanostructured device design that significantly enhances light absorption across the solar spectrum. The simulated results demonstrate that this innovative approach effectively resolves the issues of parasitic absorption, high reflection, and destructive interference, leading to a substantial enhancement in the broadband optical absorptance across the solar spectrum. This work establishes a robust framework for a-Si/PbS QD tandem solar cells, providing novel photon management design strategies and a rigorous pre-experimental verification methodology to guide future device fabrication. Therefore, this study not only diagnoses the key performance bottlenecks for this tandem cell material system but, more importantly, introduces an innovative nanostructured design. This work presents a promising approach for future performance optimization of new material systems.

## Data Availability

Data is contained within the article or Supplementary Material.

## Supplementary Materials

The following supporting information can be downloaded at https://www.mdpi.com/article/10.3390/nano16010012/s1. Note S1: Sensitivity Analysis Justifying the Point-by-Point Inversion.
