# Supplementary material for: An Experimentally Benchmarked Optical Study on Absorption Enhancement in Nanostructured a-Si/PbS Quantum Dot Tandem Solar Cells
Source: Nanomaterials (Basel). 2025 Dec 21;16(1):12. doi: 10.3390/nano16010012 (PMC12787544; doi:10.3390/nano16010012)
Supplement: Supplementary file 1 [file nanomaterials-16-00012-s001.zip › nanomaterials-4023347-supplementary.pdf]

Supplementary Materials: An Experimentally Benchmarked Optical Study on Absorption Enhancement in Nanostructured a-Si/PbS Quantum Dot Tandem Solar Cells

Qinqian Jiang [1] and Zeyu Li [1,2,*]

[1] *School of Integrated Circuits, Nanjing University of Information Science and Technology, Nanjing 210044, China*
[2] *School of Computing, Engineering & Mathematical Sciences, La Trobe University, Melbourne, VIC 3086, Australia*

* Correspondence: zeyu.li@latrobe.edu.au

Supplementary Note 1: Sensitivity Analysis Justifying the Point-by-Point Inversion

To justify the validity of our point-by-point fitting algorithm, we performed a sensitivity analysis to evaluate the impact of refractive index (n) variations—such as those imposed by Kramers-Kronig (K-K) relations—on the film's absorptance.

1. Theoretical Framework

For a thin film with complex refractive index $N = n + ik$, the reflectance (R) at normal incidence is primarily governed by the real part n:

$$R \approx \left(\frac{n-1}{n+1}\right)^2 \quad \left(\text{Assuming } k^2 \ll (n-1)^2\right) \tag{S1}$$

The absorptance (A) is governed by the absorption coefficient $\alpha = 4\pi k/\lambda$ according to Beer-Lambert's law (neglecting multi-reflection interference for simplicity in this first-order estimation):

$$A \approx (1-R)(1-e^{-\alpha d}) \tag{S2}$$

2. Sensitivity Derivation

We analyze the partial derivative of Absorptance (A) with respect to the refractive index (n):

$$\frac{\partial A}{\partial n} = \frac{\partial A}{\partial R} \cdot \frac{\partial R}{\partial n} \tag{S3}$$

The first term represents the sensitivity of absorptance to reflectance:

$$\frac{\partial A}{\partial R} = -(1-e^{-\alpha d}) \approx -1 \quad (\text{for highly absorbing media}) \tag{S4}$$

The second term represents the sensitivity of reflectance to the refractive index:

$$\frac{\partial R}{\partial n} = \frac{\partial}{\partial n}\left(\frac{n-1}{n+1}\right)^2 = \frac{4(n-1)}{(n+1)^3} \tag{S5}$$

For PbS QDs, typically $n \approx 2.5$. Substituting this value:

$$\frac{\partial R}{\partial n} \approx \frac{4(1.5)}{(3.5)^3} \approx \frac{6}{42.875} \approx 0.14 \tag{S6}$$

3. Error Estimation

The K-K relations typically induce a variation in the real part Δn_{KK} based on changes in the imaginary part. In the visible spectrum, this variation is generally small, typically $\Delta n_{KK} < 0.1$.

The resulting error in reflectance (ΔR) is:

$$\Delta R \approx \frac{\partial R}{\partial n} \cdot \Delta n_{KK} \approx 0.14 \times 0.1 = 0.014 \quad (1.4\%) \tag{S7}$$

4. Conclusion

The analysis demonstrates that a variation in n of 0.1 results in a change in reflectance of only $\sim 1.4\%$. In contrast, the experimental absorptance deviations we aim to correct are often much larger (> 5–10%).

Therefore, the absorption profile is dominantly sensitive to the extinction coefficient (k) and weakly sensitive to the refractive index (n). This confirms that fixing n while fitting k (our point-by-point method) is a mathematically robust engineering approximation that introduces negligible error into the device simulation.
